# Incongruence between Nuclear and Chloroplast DNA Phylogenies in *Pedicularis* Section *Cyathophora* (Orobanchaceae)

**DOI:** 10.1371/journal.pone.0074828

**Published:** 2013-09-19

**Authors:** Wen-Bin Yu, Pan-Hui Huang, De-Zhu Li, Hong Wang

**Affiliations:** 1 Key Laboratory of Biodiversity and Biogeography, Kunming Institute of Botany, Chinese Academy of Sciences, Kunming, Yunnan, People’s Republic of China; 2 Plant Germplasm and Genomics Center, Germplasm Bank of Wild Species, Kunming Institute of Botany, Chinese Academy of Sciences, Kunming, Yunnan, People’s Republic of China; 3 Graduate University of Chinese Academy of Sciences, Beijing, Yunnan, People’s Republic of China; J. Craig Venter Institute, United States of America

## Abstract

*Pedicularis* section *Cyathophora* is a monophyletic group characterized by perfoliate leaf and/or bract bases at each node. This section comprises four series, corresponding to four general corolla types of *Pedicularis* , i.e. toothless, toothed, beaked and long-tubed corollas. In this study, we aim to reconstruct a comprehensive phylogeny of section *Cyathophora* , and compare phylogenetic incongruence between nuclear and chloroplast datasets. Sixty-seven accessions belonging to section *Cyathophora* and 9 species for other *Pedicularis* were sampled, and one nuclear gene (nrITS) and four chloroplast genes (*matK*, *rbcL*, *trnH-psbA* and *trnL-F*) were sequenced. Phylogenetic analyses show that the topologies and networks inferred from nrITS and the concatenated chloroplast datasets were incongruent, and the nrITS phylogenies and network agreed with the morphology-based taxonomy to some degree. The chloroplast genome of two Sichuan samples of *P* *. cyathophylloides* (E4 and E5) may show introgression from an ancestor of *P* *. cyathophylla* . Neither the nrITS dataset nor the concatenated chloroplast dataset were able to adequately resolve relationships among species in the series *Reges*; this is most likely due to incomplete lineage sorting and/or introgression/hybridization. The nrITS phylogeny indicates the beakless (toothed and toothless) and beaked galeas may have evolved independently within section *Cyathophora* , and the chloroplast phylogeny reveals that the long corolla tube with beaked galea is derived from the short one.

## Introduction

**Figure 1:**
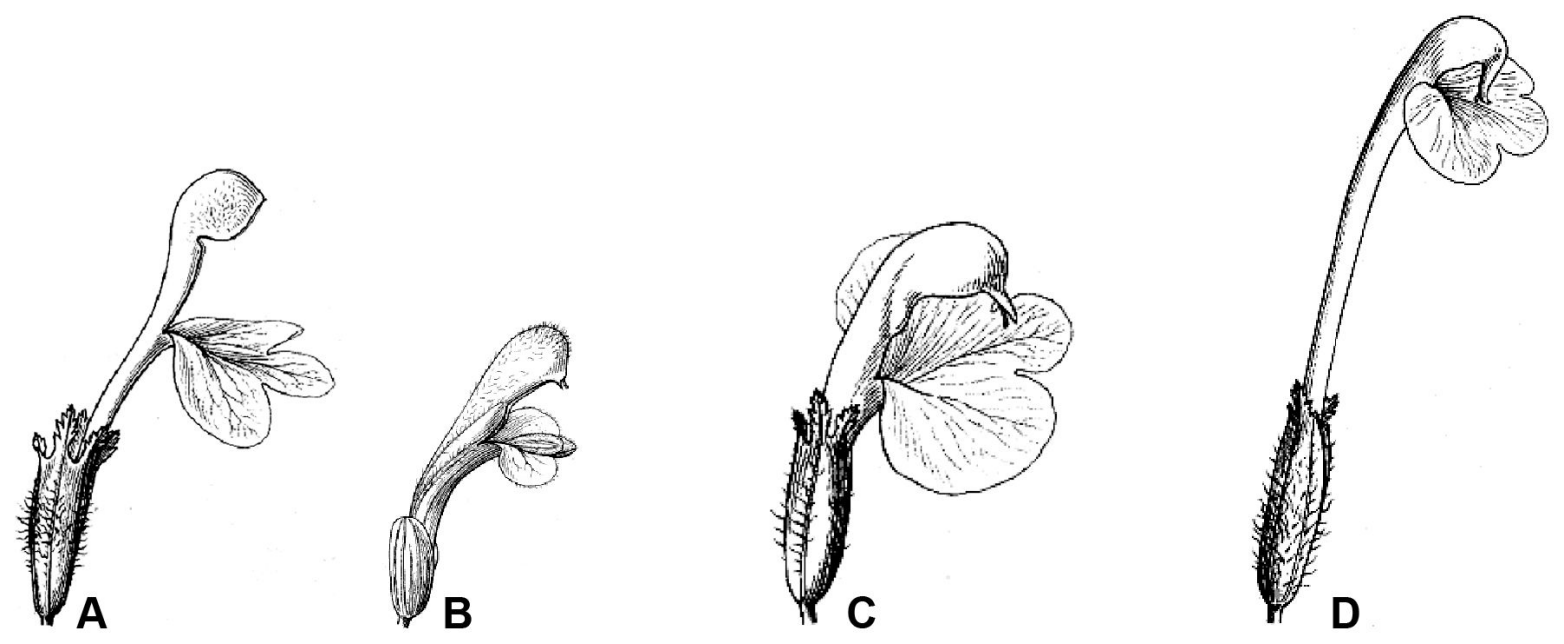
Four general corolla types in *Pedicularis* section *Cyathophora* (Redrawn from Tsoong [14]). (A) short tubular corolla with a beakless and toothless galea ( *P* *. cyathophylloides* ); (B) short tubular corolla with a toothed galea (P. *rex* subsp. *rex*); (C) short tubular corolla with a beaked galea ( *P* *. superba* ); and (D) long tubular corolla with a beaked galea ( *P* *. cyathophylla* ).

**Figure 2:**
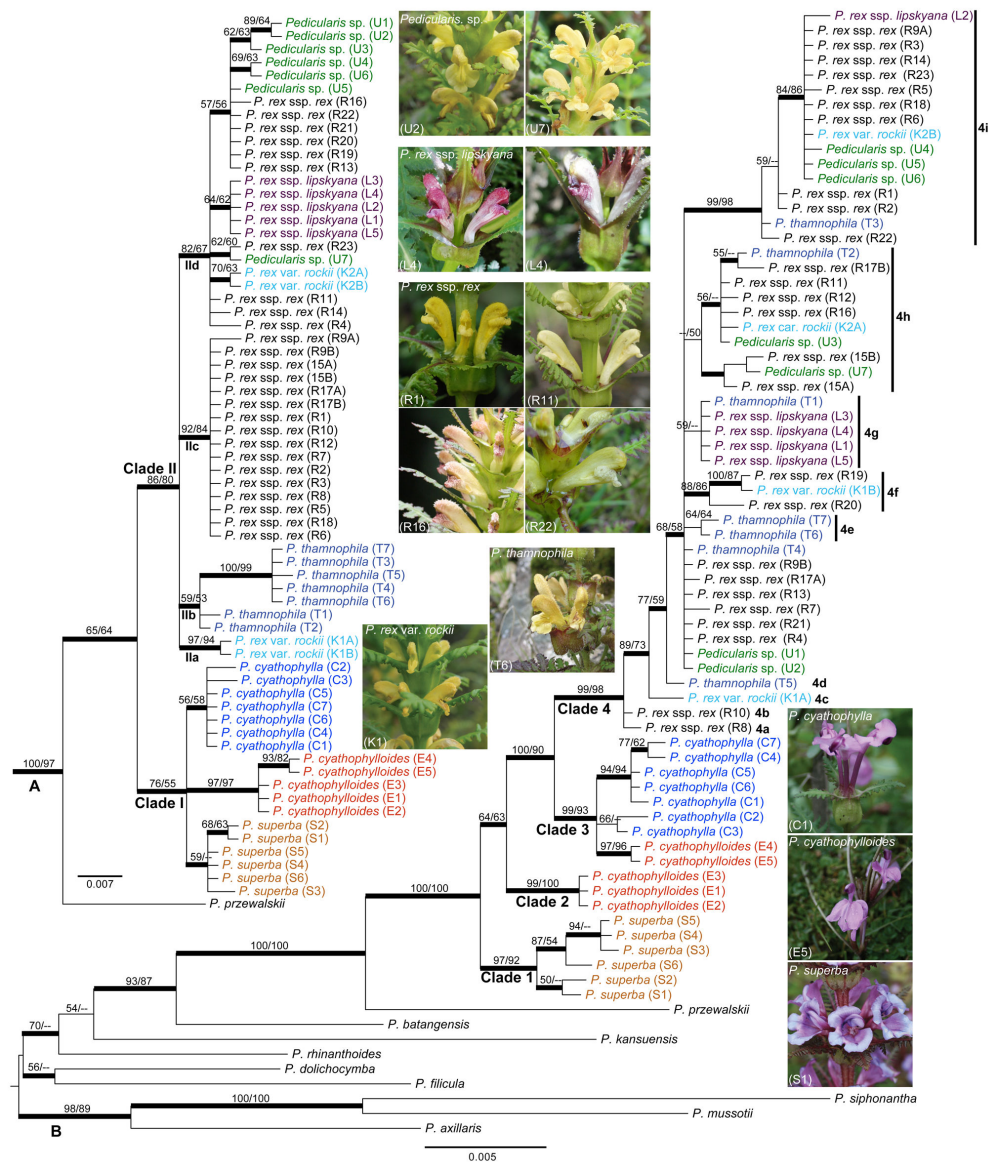
Phylogenies of *Pedicularis* section *Cyathophora* inferred from Bayesian, maximum likelihood and maximum parsimony methods using nrITS (A) and the concatenated chloroplast (B) datasets. Topology shows the majority rule consensus of the Bayesian inference tree, with thicker lines for the posterior support ≥ 0.95. Bootstrap values (≥50) of maximum likelihood/parsimony are indicated above branches. Sample code indicated in the parenthesis after species name and on the species photo is summarized in Table S1.

*Pedicularis* (Orobanchaceae) comprises approximately 600-800 species, one of the largest genera of flowering plants in the north temperate zone [1,2]. Flower morphology of *Pedicularis* exhibits great variation among species in the shape of upper corolla lip (galea) and the length of corolla tube. Species may be described as having one of four different corolla morphologies [1,3,4]: (A) short tubular corolla with beakless and a toothless galea, (B) short tubular corolla with a toothed galea, (C) short tubular corolla with a beaked galea, and (D) long tubular corolla with a beaked galea (see Figure 1). The high degree of parallel evolution in floral morphology of *Pedicularis* makes traditional infrageneric classifications controversial [5]. Noteworthily, section *Cyathophora* H. L. Li [6] is one of few monophyletic subgeneric taxa supported by molecular phylogenies [3,7,8]. This section is characterized by the bases of whorled leaves and/or bracts that are dilated and fused together to form a cup-like structure around the stem at each node (Figure 2). Despite consisting of a few species and restricted to the eastern Himalaya-Hengduan Mountains, section *Cyathophora* is highly diverse in floral shape, including all four general corolla types (Figure 1) [1,6,9].

Before the system of Li [6], authors placed the species of section *Cyathophora* in the series *Superbae* Maxim. as a cohesive unit [10-13]. Li [6] categorized eight species of section *Cyathophora* into four series in accordance with four general corolla types (Table 1). Tsoong [14] adopted the four-series classification of section *Cyathophora* by Li [6], with a revised circumscription of these series (Table 1). In addition, Tsoong [5] disagreed with Li [6] on the likely phylogenetic position of the monotypic series *Cyathophylloides* H. L. Li, which Tsoong argued was the most basal of the section He classified *P* *. cyathophylloides* as the beaked type, given its long corolla tube, swollen galea, obscure beak, and spreading lower lip. *Pedicularis* *xiangchengensis* H. P. Yang was initially placed in series *Cyathophyllae* [15], but is now considered as a synonym of *P* *. cyathophylla* Franch. [16].

**Table 1:** Overview for the treatment of section *Cyathophora* in classification system of Li [6] and Tsoong [14], and in this study.

**Sect. *Cyathophora* H. L. Li**	**System of Li (1948**)	**System of Tsoong (1963**)	**In this study**	**Corolla types**
**Ser. *Reges* H.L. Li**	*P. rex* Maxim. var. *rex* (syn.: *P* *. lopingensis* Hand.-Mazz., *P* *. mahoangensis* Bonati)	*P. rex* subsp. *Rex* (syn.: *P* *. lopingensis* Hand.-Mazz., *P* *. mahoangensis* Bonati)	*P. rex* subsp. *Rex* (syn.: *P* *. lopingensis* , *P* *. mahoangensis* , *P. rex* var. *parva*, *P. rex* var. *pseudocyathus*)	B
	*P. rex* var. *rockii* (Bonati) H.L. Li (≡*P. rockii* Bonati)	*P. rex* subsp. *rex* var. *rockii* (Bonati) H.L. Li (≡ *P* *. rockii* )	*P. rex* var. *Rockii* (≡ *P* *. rockii* Bonati)	A
	*P. rex* var. *parva* Bonati	*P. rex* subsp. *parva* (Bonati) P.C. Tsoong (≡ *P. rex* var. *parva*)		B
	*P. rex* var. *pseudocyathus* Bonati	*P. rex* subsp. *pseudocyathus* (Bonati) P.C. Tsoong (≡ *P. rex* var. *pseudocyathus*)		B
	*P* *. lipskyana* Bonati (syn.: *P* *. lamarum* Limpr.)	*P. rex* subsp. *lipskyana* (Bonati) P. C. Tsoong (≡ *P* *. lipskyana* )	*P. rex* subsp. *Lipskyana* (≡ *P* *. lipskyana* ; syn.: *P* *. lamarum* , *P. rex* subsp. *zayuensis*)	B
		*P. rex* subsp. *zayuensis* H.P. Yang (1990)		B
	*P*. *thamnophila* (Hand. -Mazz.) H. L. Li (≡ *P. rex* var. *thamnophila* Hand. -Mazz.)	*P* *. thamnophila* (≡ *P. rex* var. *thamnophila*)	*P* *. thamnophila* (≡ *P. rex* var. *thamnophila*; syn.: *P* *. cupuliformis* )	B
	*P* *. cupuliformis* H.L. Li	*P* *. thamnophila* subsp. *cupulifomis* (H.L. Li) P. C. Tsoong (≡*P. cupuliformis*)		B
**Ser. *Cyathophylloides* H. L. Li**	*P* *. cyathophylloides* Limpr.	*P* *. cyathophylloides*	*P* *. cyathophylloides*	A or C
**Ser. *Superbae* Maxim.**	*P* *. superba* Franch. ex Maxim.	*P* *. superba*	*P* *. superba*	C
		*P* *. connata*	*P* *. connata* (?)	C
**Ser. *Cyathophyllae* H. L. Li**	*P* *. connata* H.L. Li			
	*P* *. cyathophylla* Franch.	*P* *. cyathophylla* Franch.	*P* *. cyathophylla* Franch. (syn.: *P* *. xiangchengensis* )	D
		*P* *. xiangchengensis* H.P. Yang (1990)		D

Corolla types: A, short tubular corolla with a beakless and toothless galea; B, short tubular corolla with a toothed galea; C, short tubular corolla with a beaked galea; and D, long tubular corolla with a beaked galea. Question mark (?) after *P* *. connata* indicates that this taxon was not sampled in this study.

Li [6] hypothesized that the ancestral condition in section *Cyathophora* was toothless (series *Cyathophylloides*), and subsequently transformed to toothed (series *Reges*) and to beaked (series *Superbae*), culminating in the long tubular type (series *Cyathophyllae*), which is the general evolution model of floral characters in *Pedicularis* [1,6]. Tsoong [5] concurred Li’s hypothesis, whereas he placed the obscure-beaked series *Cyathophylloides* at the middle stage between series *Reges* and series *Superbae*. In addition, Tsoong [14] agreed that floral evolution of section *Cyathophora* may have begun with the toothless type, because *P. rex* var. *rockii* is toothless in series *Reges*. However, Macior [17] suspected that the extremely long-tubed and beaked *P* *. cyathophylla* was derived from the short-tubed and toothed *P. rex*. He suggested that such a saltatory evolution of floral form is possible and the gradual sequence may not represent a historical series of events. Ree [3] demonstrated that the long-tubed corolla appears to derive from a short-tubed corolla, and suggested that the pollination advantage of the beakless galea may facilitate to change from beaked to beakless in section *Cyathophora* .

Despite a long history of uncertainty regarding the evolution of floral form in section *Cyathophora* , few individuals have ever been included in previous phylogenetic reconstructions [3,7,8]. All phylogenies strongly support monophyly of section *Cyathophora* , however, relationships among its members are poorly understood. Phylogenetic incongruence was found among nuclear ribosomal internal transcribed spacer (nrITS), and chloroplast *matK* and *trnT-trnF* datasets. For example, the nrITS and *matK* datasets strongly support *P. rex* + *P* *. thamnophila* (i.e. series *Reges*) together [7,18], while the *trnT-trnF* dataset places *P. rex* sister to a clade *P* *. thamnophila* – *P* *. cyathophylla* [7]; the nrITS dataset strongly supports *P* *. cyathophylla* + *P* *. superba* together, while the *matK* dataset places *P* *. cyathophylla* sister to *P. rex* + *P* *. thamnophila* [18]. In this study, we aim to reconstruct a comprehensive phylogeny of section *Cyathophora* on the basis of extensive sampling (multiple samples for all taxa) and more genetic coverage (nrITS, *matK*, *rbcL*, *trnH-psbA* and *trnL-F*) with a specific emphasis on series *Reges*. The main goals of this analysis are to: (i) explore patterns and causes of phylogenetic incongruence between nuclear and chloroplast datasets; (ii) compare phylogenetic trees of series *Reges* with morphology-based taxonomy; and (iii) discuss evolution of floral characters in this section based on its reconstructed phylogeny.

## Materials and Methods

### Taxon sampling and ethics statement

**Figure 3:**
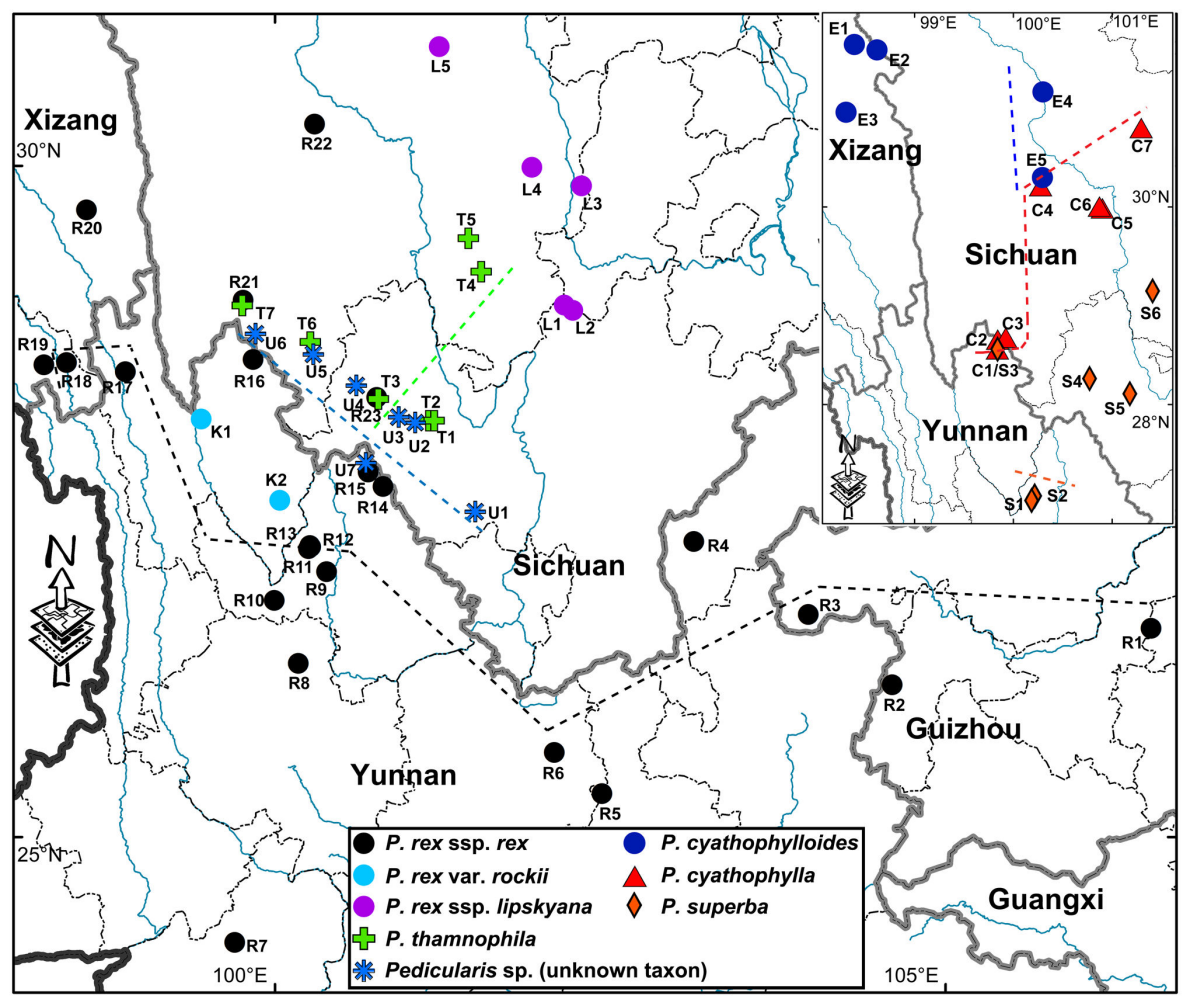
Geographical information for studied samples of *Pedicularis* section *Cyathophora* . Detailed collection of sample code is presented in Table S1. The black, green and light blue dash lines separates sample of *P*. *rex* subsp. *rex*, *P* *. thamnophila* and the unknown taxon in accordance with the nrITS phylogeny, respectively; and the blue, red and orange dash lines separates samples of *P* *. cyathophylla* , *P* *. cyathophylloides* and *P* *. superba* in accordance with the chloroplast phylogeny, respectively.

A total of 76 accessions, 67 belonging to section *Cyathophora* and 9 outgroups, were sampled in this study (Table S1). *Pedicularis* *connata* has only been collected from the type locality near Muli in southwestern China, and was not included here. No samples of endangered or protected species were included [19]. Each population of the studied species had more than several hundreds of individuals, and around three plants per population were collected as the vouchers, which were deposited in the herbarium of the Kunming Institute of Botany (KUN), Chinese Academy of Sciences. Samples were collected from public land instead of protected areas in the southwest China; therefore, field permits were not required. The morphology of vouchered specimens was compared with the type specimens as an aid in identification. *Pedicularis* *cyathophylla* , *P* *. cyathophylloides* and *P* *. superba* are easy to identify. In series *Reges*, *P* *. thamnophila* is distinguished from *P. rex* by having pilose stems, leaves and cup-like bracts, long and persistent basal leaves, a corolla lower lip spreading at right-angle to the corolla tube, and a small flower. Comparisons of the type specimens and field observations indicated that the type of *P* *. cupuliformis* consists of some short plants of *P* *. thamnophila* . In this study, therefore, we treat *P* *. cupuliformis* is conspecific with *P* *. thamnophila* . Noteworthily, we collected one unknown taxon, whose floral structure and size resemble those of *P* *. thamnophila* , whereas its stems, leaves and bracts are glabrous or sparely pubescent, and basal leaves are short or absent. For infraspecies of *P. rex*, purple/pink corollas and toothless corolla galeas were use to identify subspecies *lipskyana* and variety *rockii*, respectively. We did not accept the treatment of subspecies *parva*, *pseudocyathus* and *zayuensis* in *P. rex*, because the type specimens of the first two subspecies cannot be distinguished from specimens of the type of subspecies *rex*, and those of the third subspecies were identical to those of subspecies *lipskyana*. Herein, five species (including three infraspecific taxa for *P. rex*) (Table S1) and one unknown taxon of section *Cyathophora* were included. Nine species of *Pedicularis* were selected as outgroups. Of the list of sampled taxa is presented in Table S1, including series position, voucher information, population code and GenBank accession number. Geographical information of samples from section *Cyathophora* is indicated in Figure 3.

### Molecular methods

Total genomic DNA was extracted using a modified CTAB method [20] from silica gel dried leaves obtained from fresh field-collected specimens. One nuclear gene (nrITS) and four chloroplast genes (*matK*, *rbcL*, *trnH-psbA* and *trnL-F*) were sequenced in this study (Table 2). Protocols of PCR ampliﬁcation and sequencing were detailed in Yu et al. [21]. All new raw sequences were assembled and edited using SeqMan (DNAstar packages). Preliminary alignments were produced using MAFFT version 6.0 [22], then adjusted manually in BioEdit version 7.0 [23]. Sequence data matrixes were concatenated using SequenceMatrix version 1.7 [24].

**Table 2:** Summary of nrITS and chloroplast datasets.

	n	nrITS	Chloroplast genes					Total
			*matK*	*rbcL*	*trnH-psbA*	*trnL-F*	Combined chloroplast dataset	
Aligned length (bp)		629	1081	645	725	912	3363	3992
Variable sites/Parsimony informative sites								
Sect. *Cyathophora* + Outgroups	76	161/92	139/50	37/16	149/60	107/38	432/164	593/256
Sect. *Cyathophora*	67	46/36	30/23	11/9	29/25	19/12	89/69	135/105
Ser. *Reges*	49	28/21	14/10	2/1	19/15	9/3	44/29	72/50
*P. rex*	35	17/10	11/7	2/1	19/15	9/3	41/26	58/36
*P* *. thamnophila*	7	8/6	5/1	1/0	10/0	0/0	16/1	24/7
*P*. sp. (unknown taxa)	7	5/3	2/0	1/1	13/11	0/0	16/12	21/15
Other series	18	17/11	18/11	8/7	11/11	8/7	45/36	62/47
*P* *. cyathophylla*	7	4/0	7/2	1/1	2/2	6/0	16/5	20/5
*P* *. cyathophylloides*	5	2/2	7/7	5/5	4/4	6/3	22/19	24/21
*P* *. superba*	6	3/1	5/1	2/1	3/3	2/2	12/7	15/8

### Reconstruction of phylogenetic trees

Bayesian inference (BI), maximum likelihood (ML) and maximum parsimony (MP) methods were used to reconstruct phylogenetic trees of *Pedicularis* section *Cyathophora* . Phylogenetic resolution of the single chloroplast datasets was low, and no or low phylogenetic conflict was found among four chloroplastic genes for all three phylogenetic methods. Therefore, chloroplast loci were concatenated into a single chloroplast dataset. Chloroplast and nrITS datasets were analyzed separately. All characters were equally weighted and treated as unordered. Gaps were not coded. For BI, model selection was based on the Akaike information criterion (AIC) estimated by jModelTest [25,26]. The BI analyses were performed using MrBayes version 3.1 [27], with 10,000,000 Markov chain Monte Carlo (MCMC) generations and four incrementally heated chains. MCMC started from a random tree and sampling one of every 1000 generations, with the first 10% of the trees discarded as burn-in. The remaining trees were used to generate a majority-rule consensus tree. Consensus clades with posterior probability (PP) ≥ 0.95 are considered as strongly supported [28-32]. ML tree searches and bootstrap estimation of clade support were conducted using RAxML [33] on the CIPRES Science Gateway (http://www.phylo.org). Rate heterogeneity and proportion of invariant sites were included as model parameters, with other options left in the default settings. Support values for the node and clade were estimated from 100 bootstrap replicates. The MP analysis was conducted using PAUP* version 4.0b10 [34]. Heuristic searches were implemented with 1000 random addition sequence replicates, and tree bisection-reconnection (TBR) branch swapping with the MULTREE option. Support for the node and clade was evaluated using bootstrap analysis [35] of 1000 replicates, each with 500 random addition sequence replicates and TBR branch swapping.

### Phylogenetic network analysis

Phylogenetic networks were constructed to construct the evolutionary history of section *Cyathophora* using the SplitsTree version 4.12 [36]. The Neighbor-net model was performed using the Kimura 2-parameter (K2P) distances and Ordinary Least Square Method. The nrITS and the concatenated chloroplast datasets were analyzed separately. Bootstrap values for the respective splits were estimated from 1000 bootstrap replicates.

## Results

### Sequence characteristics

Of the 380 sequences from 76 accessions analyzed in this study (Table S1), sequences of three bar coding loci (nrITS, *rbcL* and *trnH-psbA*) for 32 accessions were previously published [21]. The remaining 284 sequences (accession numbers: KC733277-KC733352, KF011707-KF011914) were generated for this study (Table S1). Sequence characteristics of nrITS and single and concatenated chloroplast datasets are summarized in Table 2.

In the raw sequences of nrITS, ambiguous basecalls were the result of multiple superimposed peaks in chromatograms. One such base was found in five samples (E4, L2, R23, S2 and U5), two were found in three samples (R6, S5 and T7), and three were found in two sample (C1 and R5). The ambiguous site was assigned using IUPAC ambiguity characters. At the level of section and series, sequences of nrITS are more variable and informative than those of any single chloroplast gene in section *Cyathophora* and series *Reges*. Nevertheless, sequences of some chloroplast genes are more variable and informative than those of nrITS at the species level for some species. For example, *psbA-trnH* provides the most variation and parsimony informative characters in *Reges*. Similarly, *matK* provides the most variation and parsimony informative characters in *P* *. cyathophylla* and *P* *. superba* . Further, sequences of all chloroplast genes were more variable and informative than those of nrITS in *P* *. cyathophylloides* .

### Phylogenetic analysis of nrITS sequences

For nrITS dataset, monophyly of section *Cyathophora* is strongly supported using BI analysis (Figure 2A). Subsequently, section *Cyathophora* is divided into two well-supported clades. Clade II corresponds to series *Reges*, while Clade I includes the other three series, and Clade II corresponds to series *Reges*. In clade I, monophyly of *P* *. cyathophylloides* is strongly supported by all analyses, but monophyly of *P* *. cyathophylla* and *P* *. superba* is strongly-supported in BI analysis alone (Figure 2A). In addition, samples E4 and E5 form a Sichuan clade in *P* *. cyathophylloides* , and samples S1 and S2 from Lijiang in southwestern Yunnan group together in *P* *. superba* . Within the clade of series *Reges*, four subclades are identified (Figure 2A). Subclade IIa contains two samples of *P. rex* var. *rockii* from Nixi (K1) of Shageri-La in Northwestern Yunnan. Subclade IIb corresponds to *P* *. thamnophila* ; within this clade, five samples (T3-T7) from northwestern Muli of southwestern Sichuan are grouped together, and the other two samples from southeastern Muli (Changhaizi) are placed at the base of this subclade (Figure 3). Subclade IIc comprises of 16 samples of *P. rex* subsp. *rex* from the southern margin of distribution area, i.e., western Guizhou, western and central Yunnan, and Southestern Xizang (see Figure 3). Subclade IInd includes all selected infraspecific taxa of *P. rex* and all seven samples of the unknown taxon. In this subclade, two samples of *P. rex* var. *rockii* from Baishuitai (K2) of Shageri-La and all five samples of *P. rex* subsp. *lipskyana* are monophyletic, respectively. Monophyly of the unknown taxon is not supported, the seven samples occurring in four groups. With the exception of the sample ‘*LIDZ0990*’ from Yongnian (U7) of Ninglang is clustered with sample R23 of *P. rex* subsp. *rex* in a separate clade, the other six samples s occur in a poorly supported clade along with six specimens of *P. rex* ssp. *rex*, forming groups U1 + U2 + U3 and U4 + U6, and sample U5 alone.

### Phylogenetic analysis of chloroplast sequences

The monophyly of section *Cyathophora* is strongly supported by all analyses using the concatenated chloroplast dataset (Figure 2B). Within section *Cyathophora* , series *Reges* (Clade 4) and *P* *. superba* (Clade 1) are resolved as monophyletic with high support values; and five samples of *P* *. cyathophylloides* separate into two groups: three Xizang samples (E1, E2 and E3) form an independent clade, and two Sichuan samples (E4 and E5) are clustered in a clade including all accessions of *P* *. cyathophylla* (Figure 2B). *Pedicularis* *superba* (Clade 1) is sister to the remainder of section *Cyathophora* , followed by three Xizang samples of *P* *. cyathophylloides* (Clade 2). A clade including the paraphyletic clade of *P* *. cyathophylla* (Clade 3), and two (E4 and E5) of the five accessions of *P* *. cyathophylloides* is strongly supported as the sister group of series *Reges* (Clade 4) In the clade of series *Reges*, no taxon is resolved as monophyletic (Figure 2B). Samples R8 and R10 of *P. rex* spp. *rex* are placed at the base of series *Reges*, followed by K1A of *P. rex* var. *rockii* and sample T5 of *P* *. thamnophila* . The remaining samples belong to a well-supported clade; however, relationships within this clade correspond poorly with named taxa.

### Phylogenetic networks of section *Cyathophora*

**Figure 4:**
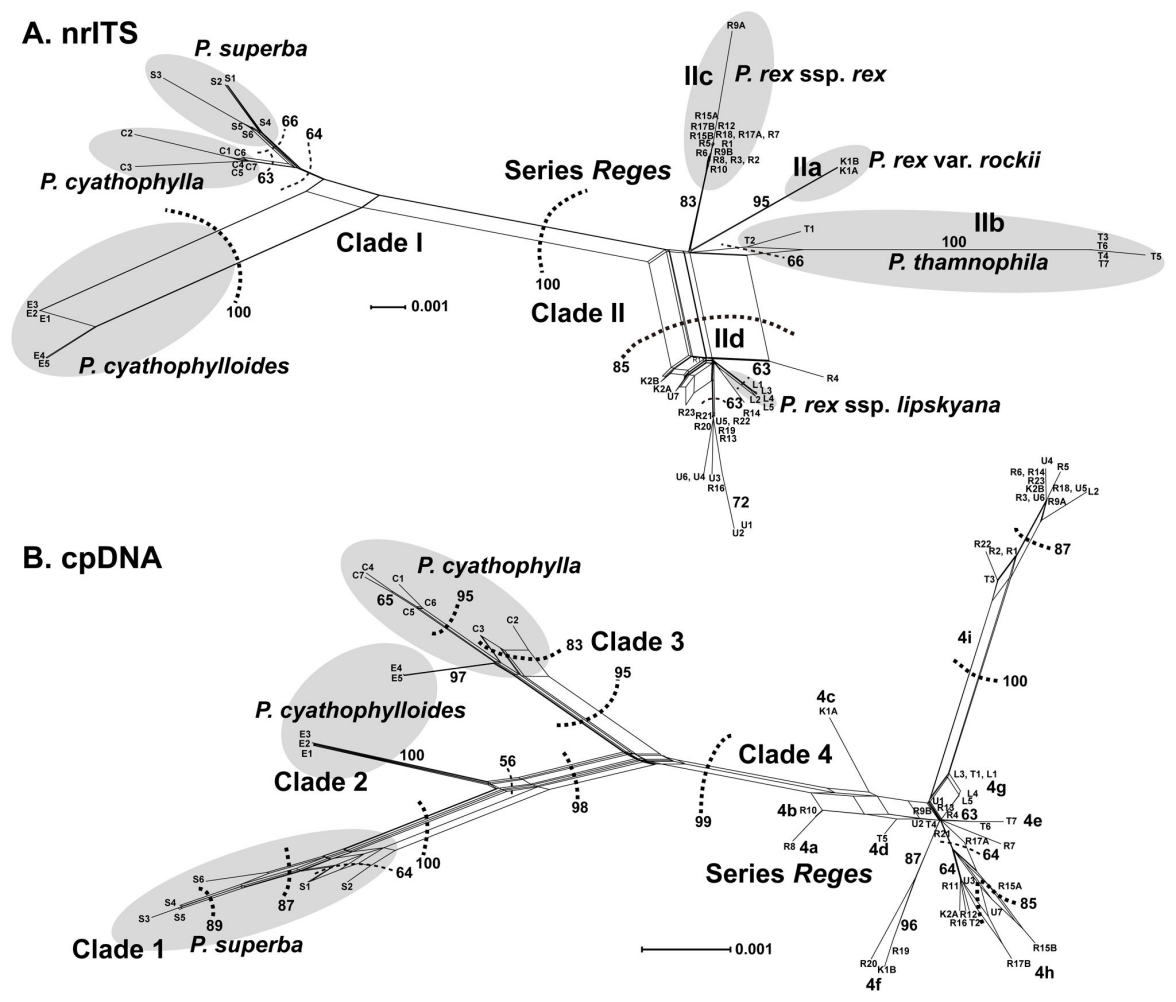
Neighbor-net analysis of *Pedicularis* section *Cyathophora* using nrITS (A) and the concatenated chloroplast (B) datasets. Bootstrap support values for clusters are indicated next to the respective cluster delimitation (dashed lines); monophyly of accepted taxa is annotated in gray, except *P* *. cyathophylloides* in the chloroplast network. Scale bar indicates changes.

Both nrITS and the concatenated chloroplast datasets split section *Cyathophora* into two major groups, series *Reges* and *P* *. cyathophylla* + *P* *. cyathophylloides* + *P* *. superba* (Figure 4). Incongruence between nrITS and the chloroplast networks was evident. The nrITS placed *P* *. cyathophylloides* close to *P* *. cyathophylla* + *P* *. superba* , whereas the chloroplast dataset split *cyathophylloides* into two groups, two Sichuan samples (E4 and E5) grouped with *P* *. cyathophylla* , and three Xizang samples (E1, E2 and E3) close to *P* *. superba* . Within the series *Reges* subnetwork of the nrITS analysis, monophyletic groups of *P. rex* subsp. *lipskyana*, subsp. *rex* and *P* *. thamnophila* form subnetworks of their own, while none of these groups occur in the chloroplast network.

## Discussion

### Informativeness of DNA markers

The nrITS region has been screened multiple times in previous studies of *Pedicularis* , and it is in general the most informative DNA marker for phylogeny [3,7,8] and species discrimination [21] in *Pedicularis* to date. Within section *Cyathophora* , the nrITS dataset also shows greater polymorphism than the other four screened chloroplast markers. Curiously, intraspecific sequences variation of *trnH-psbA* are higher than those of nrITS in all species, and those of all four chloroplast markers are higher than those of nrITS in *P* *. cyathophylloides* . Our results show that *trnH-psbA* sequences have high rates of intra-specific insertion/deletion [21], which may make difficult to align for large scale phylogeny of *Pedicularis* . The high amount of polymorphisms in the chloroplast markers of *P* *. cyathophylloides* results from polyphyletic relationships among five samples. The *rbcL* gene shows low variation within the genus *Pedicularis* [21], however, it does provide more informative sites than *trnH-psbA* and *trnL-F* in *P* *. cyathophylloides* , which indicating that the chloroplast genomes of two subclades in *P* *. cyathophylloides* may be heterogenous.

### Incongruence between nuclear and chloroplast DNA phylogenies

Monophyly of section *Cyathophora* is well supported by all phylogenetic analyses in this study, as well as in previous studies [3,7,8]. Furthermore, monophyly of series *Reges* is strongly supported by all analyses. We did not concatenate nrITS and chloroplast datasets to infer phylogenies, because phylogenetic topologies between nrITS and the concatenated chloroplast datasets are heavily incongruent (see Figure 2). The only strongly congruent results between separate analysis of plastid and nuclear DNA are: (i) series *Reges* is monophyletic in both analyses (although no taxon is supported as monophyletic in either analysis), and (ii) all samples of *P* *. superba* form clades in both analyses. The chloroplast phylogeny was incongruent with morphology-based taxonomy, except *P* *. superba* , whereas the nrITS phylogenies agreed with the morphology-based taxonomy to some degree. The nrITS phylogeny successfully delimits four species and one infraspecific taxon as monophyletic: *P* *. cyathophylloides* , *P* *. cyathophylla* , *P* *. superba* and *P* *. thamnophila* , and *P. rex* spp. *lipskyana*. Furthermore, the nrITS phylogeny supports the contentions of Tsoong [5] that *P* *. cyathophylloides* is close to *P* *. cyathophylla* and *P* *. superba* , a result that has also been supported by pollen morphology [9]. In addition, nrITS sequences of the sample ‘*HW10215*’ of *P* *. cyathophylla* (C3) collected from the type locality of ‘ *P* *. xiangchengensis* ’ [15] is indistinct from the other six samples of *P* *. cyathophylla* in agreement with previous morphological comparisons [16]. Our results support the prediction of Petit and Excoffier [37] that biparentally inherited nuclear loci experiencing high rates of intraspecific gene flow should enhance species delimitation, and that maternally inherited chloroplast loci should be more frequently introgressed and hence of more limited use in species delimitation than nuclear loci. In addition, incomplete lineage sorting in the slower evolving chloroplast genes may cause phylogeny of chloroplastic gene incongruent with that of nuclear gene and the morphology-based taxonomy in section *Cyathophora* [38-40].

### Chloroplast capture in P. *cyathophylloides*

Incongruence between nuclear and chloroplast phylogenies might be explained as convergent evolution, lineage sorting, or reticulate evolution [39,41-43]. However, such processes cannot explain the incongruence in the placement of *P* *. cyathophylloides* . Chloroplast phylogenies separate five samples of *P* *. cyathophylloides* into two groups, two Sichuan samples and three Xiang samples, and the Sichuan group falls into the clade *P* *. cyathophylla* . Haplotypes did not vary within sites, evidence of strong geographical partitioning. Herein, we suggest that chloroplast capture, the introgression of a chloroplast from one species into another following a hybridization event followed by backcrossing of F1s with parental types, may be the most likely explanation for the pattern observed here [44-46]. The geographical pattern indicates that the chloroplast of the Sichuan group of *P* *. cyathophylloides* may be introgressed from that of *P* *. cyathophylla* . Collection records show that the distributions of *P* *. cyathophylloides* and *P* *. cyathophylla* overlap in western Sichuan. Hybridization may be mediated by bumblebee pollinators. However, the nrITS phylogenies clearly reject the recent hybridization between two species. Therefore, chloroplast capture in *P* *. cyathophylloides* is likely the result of an ancient hybridization/introgression event; and the current overlapping distribution may be a region the secondary contact region, in which evolution of floral character displacement effectively mediates reproductive isolation between two species.

### Phylogenetic implications in series Reges

Series *Reges* is a monophyletic group supported by all analyses in this study and it is characterized by a yellow corolla (except *P. rex* subsp. *lipskyana*), short corolla tube, toothed galea (except *P. rex* var. *rockii*) and small lower lip. The nrITS phylogenies partially support the morphology-based taxonomic treatment in series *Reges*. While the number of phylogenetically informative sites and clade resolution of the concatenated chloroplast dataset are higher than those of nrITS dataset in series *Reges*, none of the morphology-based taxa is resolved in the chloroplast phylogeny. Even two individuals from the same population of *P. rex* subsp. *rex* (R9 and R17) and of *P. rex* var. *rockii* (K1 and K2) occur in different subclades of Clade 4. Phylogenetic incongruence with morphology-based taxonomy in series *Reges* may be caused by incomplete lineage sorting [40] and/or hybridization/introgression [37,42,47-49], and/or other biological factors [39,44,46,50-52].

In the nrITS phylogenies, taxonomical treatments for *P* *. thamnophila* and *P. rex* subsp. *lipskyana* are supported, while *P. rex* spp. *rex* and *P. rex* var. *rockii* are polyphyletic. Noteworthily, the subclade IInd of the nrITS phylogeny contains all recognized infraspecific taxa of *P. rex* and the unknown taxon, which are overlapped in the Hengduan Mountains (see Figure 3). By contrary, samples of *P. rex* subsp. *rex* from south margins of distribution range are fallen into the subclade IIc. The phylogenetic network shows that evolutionary history of subclade IInd was more complicated than that of other subclades. We suggested that paraphily of *P. rex* spp. *rex* may be explained by incomplete lineage sorting during population expansion, and polyphyly of *P. rex* var. *rockii* may be caused by ancient hybridization/introgression or convergent evolution [53]. The nrITS dataset shows that the unknown taxon is close to *P. rex*, than to *P* *. thamnophila* , and the sample ‘*LIDZ0990*’ (U7) is distinct from its other six samples, most closely related to the sample ‘*LIDZ1011*’ of *P. rex* subsp. *rex* (R23). Morphologically, specimens of the sample ‘*LIDZ0990*’ have a spreading lower lip that resembles closely that of the unknown taxon, however, their dense inflorescences and large corollas [54] resemble those of *P. rex* subsp. *rex*. Both morphology and nrITS phylogeny imply that the sample ‘*LIDZ0990*’ may be an evolutionary intermediate or a hybrid between *P. rex* subsp. *rex* and the unknown taxon.

### Phylogeographic patterns in section *Cyathophora*

Phylogenetic relationships are correlated with geographic distribution in *P. rex* subsp. *rex* and var. *rockii* and *P* *. thamnophila* using nrITS dataset, and in *P* *. cyathophylla* , *P. cyathophylloides*a and *P* *. superba* using chloroplast dataset. In the nrITS phylogeny, samples of *P. rex* subsp. *rex* from the south margin of the distribution occur in subclade IIc, while the remaining samples are included in subclade IInd. This pattern indicates that southern samples of *P. rex* subsp. *rex* may still be subject to gene flow, or may have rapidly expanded in recent years. Meanwhile, the most recent common ancestor (MRCA) of the northern samples in subclade IInd may have experienced rapid species divergence, with four other taxa derived from this MRCA. Four samples of *P. rex* var. *rockii* occur in two widely separated subclades corresponding with geographic localities. This pattern indicates that gene flow between two populations in this taxon is lower, and samples of K2 may have gene exchange with the other taxa in the subclade IInd. For *P* *. thamnophila* , five samples from northwestern Muli (T3-T7) are separated from samples T1 and T2 in southeastern Muli by a long branch with strong support, indicating gene flow among the five northwestern samples is stronger than that between the two groups, or the five northwestern samples may be derived from a MRCA that separated from the MRCA of samples T1/T2 and rapidly expanded in recent years.

In the chloroplast phylogenies, the phylogeography of *P* *. cyathophylloides* is likely caused by chloroplast capture in two Sichuan samples (E4 and E5) (see above). Incidentally, the nrITS phylogeny also supports samples E4 and E5 together. For *P* *. cyathophylla* , three southwestern samples in Daxueshan are divided into two groups: two samples (C2 and C3) group together, while sample C1 is clustered with the other four northeastern samples (C4 ‒ C7). The geographic pattern of *P* *. superba* is close to that of *P* *. cyathophylla* . Two southwestern samples (S1 and S2) from Lijiang, Yunnan are isolated from the other three northeastern samples collected in Muli and Jiulong of Sichuan.

The geographic distribution of maternally inherited chloroplast haplotypes provides key evidence for investigating history of migration and population expansion at species level. Several phylogeographic studies have documented that the south margin of the Hengduan Mountains was one of the major glacial refugia for high altitude adapted during ice-age cycles of the Quaternary period [55-58]. In this study, phylogenetic networks inferred from both plastid and nuclear datasets indicate that evolutionary lineages of samples C2 and C3 of *P* *. cyathophylla* and samples S1 and S2 of *P* *. superba* may be the earliest-derived in their respective lineages. Therefore, Yulongshan in Lijiang and Daxueshan in the boundary of Yunnan and Sichuan may be or be close to the origin/refuge for *P* *. cyathophylla* and *P* *. superba* , respectively. For series *Reges*, phylogenetic topology and network do not support any morphology-based taxa and geographical pattern. Reticulate evolution of this group suggests that incomplete lineage sorting and/or hybridization/introgression may affect all taxa of series *Reges*.

### Evolution of floral characters in section *Cyathophora*

The nrITS phylogeny shows that evolution of the beakless (toothed and toothless) and beaked galeas are independent within section *Cyathophora* , and the long corolla tube is associated with the beaked galea. The concatenated chloroplast phylogeny reveals that the long corolla tube is derived from a short corolla tube, and the beakless galea in series *Reges* may be inherited from that of the common ancestor or be reversed from the beaked one. Ree [3] suggested that pollination advantage of the beakless galea in series *Reges* may have facilitated its geographic expansion in the Hengduan Mountains. Field observations show that *P. rex* and *P* *. thamnophila* reward bumblebees and *Amegilla* sp. with both nectar and pollen [54,59,60]. On the contrary, bumblebees forage *P* *. cyathophylla* and *P* *. superba* for pollen alone [61; W.-B. Yu unpublished data]. Morphologically, the nectary of both *P. rex* and *P* *. thamnophila* is virgate at the base of the ovary, whereas that of *P* *. cyathophylla* and *P* *. superba* is absent or indistinct (H. Wang, unpublished data). Loss of nectar production may be associated with evolution of a long corolla tube and beaked galea [3]. Pollen morphology suggests that the bisyncolpate aperture in series *Superbae* and *Cyathophyllae* may be derived from the trisyncolpate aperture in *P* *. cyathophlloides* and/or series *Reges* [9,62]. Both nectar production and trisyncolpate aperture may be plesiomorphic in *Pedicularis* , indicating that the beakless galea in series *Reges* may also be plesiomorphic.

*Pedicularis* *rex* includes both toothed and toothless varieties: subspecies *rex* and *lipskyana* are toothed, and variety *rockii* is toothless. Due to the smaller corollas of variety *rockii*, Li [6] suggested that the changed from a toothed galea to a toothless galea might have resulted from diminishing the galea size. Nevertheless, Tsoong [14] considered that the the toothless galea of variety *rockii* may be a reversal. Recently, stochastic mapping has shown that the derived toothed galea is rarely reversed to toothless galea at the genus level [3]. In this study, the nrITS phylogeny indicates that the toothless variety *rockii* may be independent from other of taxa in series *Reges*, whereas the concatenated chloroplast phylogeny suggests that the toothless variety *rockii* may be derived from the toothed subspecies *rex*. However, phylogenetic incongruence does not allow a straightforward interpretation. Noteworthily, morphometric analyses in series *Reges* show that galea size of *P. rex* var *rockii* is the smallest, followed by that of *P* *. thamnophila* and the unknown taxon (excluding *LIDZ1990*), and that the galea of subspecies *rex* and *lipskyana* of *P. rex* is the largest [54]. Morphologically, the galea teeth of *P* *. thamnophila* and the unknown taxon (excluding *LIDZ1990*) are smaller than those of subspecies *rex* and *lipskyana* of *P. rex* (see Figure 2). If the galea teeth of variety *rockii* have been reduced [6], the evidence of galea teeth can be revealed by investigating the galea development [e.g. 63] in a further study.

## Supporting Information

Table S1 **Summary information of included samples in this study.**
(DOC)
