# Supplementary material for: Incongruence between Nuclear and Chloroplast DNA Phylogenies in Pedicularis Section Cyathophora (Orobanchaceae)
Source: PLoS One. 2013 Sep 19;8(9):e74828. doi: 10.1371/journal.pone.0074828 (PMC3777957; doi:10.1371/journal.pone.0074828)
Supplement: Table S1 — Summary information of included samples in this study. (DOC) [file pone.0074828.s001.doc]

**Table S2.** Summary information of included samples in this study. New sequences (KC733277-KC733352, KF011707-KF011914) are annotated by the bold style.

| Taxon | Code | Voucher | Locality | nrITS | *matK* | *rbcL* | *trnH-psbA* | *trnL-F* |
| --- | --- | --- | --- | --- | --- | --- | --- | --- |
| Sect. *Cyathophora* H. L. Li |  |  |  |  |  |  |  |  |
| Ser. *Cyathophyllae* H. L. Li |  |  |  |  |  |  |  |  |
| *P. cyathophylla* Franch. | C1 | W.-B. Yu et al. LIDZ1268 | Daxeushan, Shangrila,Yunnan | JF977518 | **KF011847** | JF943001 | JN045934 | **KC733332** |
|  | C2 | W.-B. Yu et al. HW10208 | Daxueshan, Xiangcheng, Sichuan | **KF011707** | **KF011841** | **KF011751** | **KF011795** | **KC733326** |
|  | C3 | W.-B. Yu et al. HW10215 | Daxueshan, Xiangcheng, Sichuan | JF977517 | **KF011842** | JF943000 | JN045933 | **KC733327** |
|  | C4 | W.-B. Yu et al. LIDZ1204 | Junba, Litang, Sichuan | JF977519 | **KF011846** | JF943002 | JN045935 | **KC733331** |
|  | C5 | W.-B. Yu et al. LIDZ1170 | Jianziwanshan, Yajiang, Sichuan | **KF011708** | **KF011843** | **KF011752** | **KF011796** | **KC733328** |
|  | C6 | W.-B. Yu et al. LIDZ1198 | Jianziwanshan Yajiang, Sichuan, | JF977520 | **KF011845** | JF943003 | JN045936 | **KC733330** |
|  | C7 | W.-B. Yu et al. LIDZ1180 | Geka, Daofu, Sichuan | JF977521 | **KF011844** | JF943004 | JN045937 | **KC733329** |
| Ser. *Cyathophylloides* H. L. Li |  |  |  |  |  |  |  |  |
|  | E1 | D.E. Bourfford et al. 41954 | Gantuo, Jiangda , Xizang | JF977522 | **KF011850** | JF943005 | JN045938 | **KC733335** |
|  | E2 | W.-B. Yu et al. YWB132 | Gangtuo, Jiangda, Xizang | JF977526 | **KF011852** | JF943009 | JN045942 | **KC733337** |
|  | E3 | D.E. Bourfford et al. 41478 | Xiangpi, Gongjue, Xizang | JF977523 | **KF011849** | JF943006 | JN045939 | **KC733334** |
|  | E4 | D.E. Bourfford et al. 36271 | Le'an, Xinlong, Sichuan | JF977524 | **KF011848** | JF943007 | JN045940 | **KC733333** |
|  | E5 | W.-B. Yu et al. LIDZ1194 | Junba, Litang, Sichuan | JF977525 | **KF011851** | JF943008 | JN045941 | **KC733336** |
| Ser. *Reges* H. L. Li |  |  |  |  |  |  |  |  |
| *P. rex* C. B. Clarke subsp. *rex* | R1 | S.-D. Zhang 20090356 | Qingzhen, Guizhou | **KF011715** | **KF011863** | **KF011759** | **KF011803** | **KC733295** |
|  | R2 | W.-B. Yu et al. LIDZ0962 | Lidiping, Panxian, Guizhou | JF977684 | **KF011869** | JF943167 | JN046098 | **KC733301** |
|  | R3 | W.-B. Yu et al. LIDZ0979 | Heishitou, Weining, Guizhou | JF977683 | **KF011870** | JF943166 | JN046097 | **KC733302** |
|  | R4 | Z.-X. Ren s.n. | Yaoshan, Qiaojia, Yunnan | **KF011730** | **KF011883** | **KF011774** | **KF011818** | **KC733311** |
|  | R5 | W.-B. Yu et al. LIDZ1300 | Laoqingshan, Fumin, Yunnan | **KF011727** | **KF011880** | **KF011771** | **KF011815** | **KC733308** |
|  | R6 | H. Wang s.n. | Wuding, Yunnan | **KF011731** | **KF011884** | **KF011775** | **KF011819** | **KC733312** |
|  | R7 | W. Jiang J118 | Wumulong, Yongde, Yunnan | **KF011720** | **KF011868** | **KF011764** | **KF011808** | **KC733300** |
|  | R8 | W.-B. Yu et al. LIDZ0983 | Jumachang, Eryuan, Yunnan | JF977682 | **KF011871** | JF943165 | JN046096 | **KC733303** |
|  | R9A | W.-B. Yu et al. LIDZ0984A | Dongjiang, Lijiang, Yunnan | **KF011721** | **KF011872** | **KF011765** | **KF011809** | **KC733289** |
|  | R9B | W.-B. Yu et al. LIDZ0984B | Dongjiang, Lijiang, Yunnan | **KF011722** | **KF011873** | **KF011766** | **KF011810** | **KC733290** |
|  | R10 | W.-B. Yu et al. HW10047 | Jiuhe, Yulong, Yunnan | **KF011716** | **KF011864** | **KF011760** | **KF011804** | **KC733296** |
|  | R11 | W.-B. Yu et al. HW10069 | Yulongshan, Yulong, Yunnan | **KF011717** | **KF011865** | **KF011761** | **KF011805** | **KC733297** |
|  | R12 | W.-B. Yu et al. HW10071 | Yulongshan, Lijiang, Yunnan | **KF011718** | **KF011866** | **KF011762** | **KF011806** | **KC733298** |
|  | R13 | W.-B. Yu et al. HW10074 | Yulongshan, Lijiang, Yunnan | **KF011719** | **KF011867** | **KF011763** | **KF011807** | **KC733299** |
|  | R14 | W.-B. Yu et al. LIDZ0988 | Luguhu, Ninglang, Yunnan | **KF011723** | **KF011874** | **KF011767** | **KF011811** | **KC733304** |
|  | R15A | W.-B. Yu et al. LIDZ0989A | Yongning, Ninglang, Yunnan | **KF011724** | **KF011875** | **KF011768** | **KF011812** | **KC733291** |
|  | R15B | W.-B. Yu et al. LIDZ0989B | Yongning, Ninglang, Yunnan | **KF011725** | **KF011876** | **KF011769** | **KF011813** | **KC733292** |
|  | R16 | W.-B. Yu et al. LIDZ1276 | Daxueshan, Shangrila, Yunnan | **KF011726** | **KF011879** | **KF011770** | **KF011814** | **KC733307** |
|  | R17A | W.-B. Yu et al. YWB163A | Feilaisi, Deqin, Yunnan | **KF011734** | **KF011887** | **KF011778** | **KF011822** | **KC733293** |
|  | R17B | W.-B. Yu et al. YWB163B | Feilaisi, Deqin, Yunnan | **KF011735** | **KF011888** | **KF011779** | **KF011823** | **KC733294** |
|  | R18 | S.-D. Zhang STET158 | Chawalong, Chayu, Xizang | **KF011732** | **KF011885** | **KF011776** | **KF011820** | **KC733313** |
|  | R19 | S.-D. Zhang STET570 | Chawalong, Chayu, Xizang | **KF011733** | **KF011886** | **KF011777** | **KF011821** | **KC733314** |
|  | R20 | L. Lu LL07001 | Yanjing, Mangkang, Xizang | **KF011729** | **KF011882** | **KF011773** | **KF011817** | **KC733310** |
|  | R21 | W.-B. Yu et al. LIDZ1284 | Rerangong, Xiangcheng, Sichuan | **KF011728** | **KF011881** | **KF011772** | **KF011816** | **KC733309** |
|  | R22 | W.-B. Yu et al. LIDZ1193 | Junba, Litang, Sichuan | JF977680 | **KF011878** | JF943163 | JN046094 | **KC733306** |
|  | R23 | W.-B. Yu et al. LIDZ1011 | Shuiluo, Muli, Sichuan | JF977681 | **KF011877** | JF943164 | JN046095 | **KC733305** |
| *P. rex* var. *rockii* (Bonati) H.L. Li | K1A | W.-B. Yu et al. LIDZ1282A | Nixi, Shangrila, Yunnan | **KF011738** | **KF011891** | **KF011782** | **KF011826** | **KC733317** |
|  | K1B | W.-B. Yu et al. LIDZ1282B | Nixi, Shangrila, Yunnan | **KF011739** | **KF011892** | **KF011783** | **KF011827** | **KC733318** |
|  | K2A | L.-M. Gao et al. 081113A | Sanba, Shangrila, Yunnan | **KF011736** | **KF011889** | **KF011780** | **KF011824** | **KC733315** |
|  | K2B | L.-M. Gao et al. 081113B | Sanba, Shangrila, Yunnan | **KF011737** | **KF011890** | **KF011781** | **KF011825** | **KC733316** |
| *P. rex* subsp. *lipskyana* (Bonati) P.C. Tsoong | L1 | W.-B. Yu et al. LIDZ1060 | Yele, Mianning, Sichuan | **KF011713** | **KF011861** | **KF011757** | **KF011801** | **KC733287** |
|  | L2 | W.-B. Yu et al. HW10360 | Liziping, Shimian, Sichuan | **KF011712** | **KF011860** | **KF011756** | **KF011800** | **KC733286** |
|  | L3 | W.-B. Yu et al. HW10342 | Erlangshan, Luding, Sichuan | **KF011710** | **KF011858** | **KF011754** | **KF011797** | **KC733284** |
|  | L4 | W.-B. Yu et al. HW10346 | Zheduotan, Kangding, Sichuan | **KF011711** | **KF011859** | **KF011755** | **KF011799** | **KC733285** |
|  | L5 | W.-B. Yu et al. LIDZ1126 | Geka, Daofu,Sichuan | **KF011714** | **KF011862** | **KF011758** | **KF011802** | **KC733288** |
| *P. thamnophila* (Hand. -Mazz.) Li | T1 | W.-B. Yu et al. LIDZ1002 | Changhaizi, Muli, Sichuan | JF977751 | **KF011909** | JF943234 | JN046164 | **KC733279** |
|  | T2 | W.-B. Yu et al. LIDZ1006 | Changhaizi, Muli, Sichuan | **KF011746** | **KF011910** | **KF011790** | **KF011834** | **KC733280** |
|  | T3 | W.-B. Yu et al. LIDZ1027 | Shuiluo, Muli, Sichuan | **KF011747** | **KF011911** | **KF011791** | **KF011835** | **KC733281** |
|  | T4 | W.-B. Yu et al. LIDZ1120 | Tanggu, Jiulong, Sichuan | **KF011749** | **KF011913** | **KF011792** | **KF011836** | **KC733277** |
|  | T5 | W.-B. Yu et al. LIDZ1101 | Liuba, Kangding, Sichuan | **KF011748** | **KF011912** | **KF011793** | **KF011837** | **KC733282** |
|  | T6 | W.-B. Yu et al. LIDZ1250 | Chitu, Daocheng, Sichuan | **KF011750** | **KF011914** | **KF011794** | **KF011838** | **KC733283** |
|  | T7 | W.-B. Yu et al. HW10234 | Reranggong, Xiangcheng, Sichuan | **KF011745** | **KF011908** | **KF011789** | **KF011833** | **KC733278** |
| *Pedicularis* sp. | U1 | W.-B. Yu et al. LIDZ1014 | Yanjing, Muli, Sichuan | JF977754 | **KF011896** | JF943237 | JN046167 | **KC733320** |
|  | U2 | W.-B. Yu et al. LIDZ1016 | Ke'er, Muli, Sichuan | JF977753 | **KF011897** | JF943236 | JN046166 | **KC733321** |
|  | U3 | W.-B. Yu et al. LIDZ1019 | Boke, Muli, Sichuan | JF977752 | **KF011898** | JF943235 | JN046165 | **KC733322** |
|  | U4 | W.-B. Yu et al. LIDZ1034 | Shuiluo, Muli, Sichuan | **KF011740** | **KF011899** | **KF011784** | **KF011828** | **KC733323** |
|  | U5 | W.-B. Yu et al. LIDZ1243 | Chitu, Daocheng, Sichuan | **KF011741** | **KF011900** | **KF011785** | **KF011829** | **KC733324** |
|  | U6 | W.-B. Yu et al. LIDZ1277 | Daxueshan, Xiangcheng, Sichuan | **KF011742** | **KF011901** | **KF011786** | **KF011830** | **KC733325** |
|  | U7 | W.-B. Yu et al. LIDZ0990 | Yongning, Ninglang, Yunnan | JF977755 | **KF011895** | JF943238 | JN046168 | **KC733319** |
| Ser. *Superbae* Maxim. |  |  |  |  |  |  |  |  |
|  | S1 | W.-B. Yu et al. LIDZ1578 | Yulongshan, Yulong, Yunnan | JF977739 | **KF011907** | JF943222 | JN046153 | **KC733343** |
|  | S2 | W. Jiang 0871 | Yulongshan, Yulong, Yunnan | **KF011743** | **KF011902** | **KF011787** | **KF011831** | **KC733338** |
|  | S3 | W.-B. Yu et al. LIDZ1266 | Daxueshan, Shangrila, Yunnan | JF977740 | **KF011906** | JF943223 | JN046154 | **KC733342** |
|  | S4 | W.-B. Yu et al. LIDZ1037 | Shuiluo, Muli, Sichuan | **KF011744** | **KF011904** | **KF011788** | **KF011832** | **KC733340** |
|  | S5 | W.-B. Yu et al. LIDZ1007 | Changhaizi, Muli, Sichuan | JF977742 | **KF011903** | JF943225 | JN046156 | **KC733339** |
|  | S6 | W.-B. Yu et al. LIDZ1074 | Wuxuhu, Jiulong, Sichuan | JF977741 | **KF011905** | JF943224 | JN046155 | **KC733341** |
| Outgroups |  |  |  |  |  |  |  |  |
| *P. axillaris* Franch. |  | W.-B. Yu et al. LIDZ1290 | Daxueshan, Xiangcheng, Sichuan | JF977478 | **KF011839** | JF942961 | JN045896 | **KC733345** |
| *P. batangensis* Bur. et Franch. |  | W.-B. Yu et al. LIDZ1067 | Jiulong, Sichuan | JF977482 | **KF011840** | JF942965 | JN045900 | **KC733346** |
| *P. dolichocymba* Hand. -Mazz. |  | S.-D. Zhang 08827 | Baimashan, Deqin, Yunnan | JF977554 | **KF011853** | JF943037 | JN045970 | **KC733347** |
| *P. filicula* Franch. |  | W.-B. Yu et al. LIDZ1520 | Jiaozishan, Dongchuan, Yunnan | **KF011709** | **KF011854** | **KF011753** | **KF011797** | **KC733348** |
| *P. kansuensis* Maxim. |  | W.-B. Yu et al. LIDZ1111 | Tanggu, Jiulong, Sichuan | JF977596 | **KF011855** | JF943079 | JN046012 | **KC733349** |
| *P. mussotii* Franch. |  | W.-B. Yu et al. LIDZ1088 | Zheduoshan, Kangding, Sichuan | JF977647 | **KF011856** | JF943130 | JN046061 | **KC733350** |
| *P. przewalskii* Maxim. |  | W.-B. Yu et al. LIDZ1235 | Wumingshan, Xiangcheng,Sichuan | JF977666 | **KF011857** | JF943149 | JN046080 | **KC733344** |
| *P. rhinanthoides* Schrenk |  | S.-D. Zhang 08938 | Bangda, Basu, Xizang | JF977687 | **KF011893** | JF943170 | JN046101 | **KC733352** |
| *P. siphonantha* Franch. |  | W.-B. Yu et al. LIDZ1580 | Yulongshan, Yulong, Yunnan | JF977725 | **KF011894** | JF943208 | JN046139 | **KC733351** |
